# *Vickermania* gen. nov., trypanosomatids that use two joined flagella to resist midgut peristaltic flow within the fly host

**DOI:** 10.1186/s12915-020-00916-y

**Published:** 2020-12-02

**Authors:** Alexei Y. Kostygov, Alexander O. Frolov, Marina N. Malysheva, Anna I. Ganyukova, Lyudmila V. Chistyakova, Daria Tashyreva, Martina Tesařová, Viktoria V. Spodareva, Jana Režnarová, Diego H. Macedo, Anzhelika Butenko, Claudia M. d’Avila-Levy, Julius Lukeš, Vyacheslav Yurchenko

**Affiliations:** 1 Life Science Research Centre, Faculty of Science, University of Ostrava, Chittussiho 10, 710 00 Ostrava, Czechia; 2 Zoological Institute of the Russian Academy of Sciences, St. Petersburg, 199034 Russia; 3 Institute of Parasitology, Czech Academy of Sciences, 370 05 České Budějovice, Czechia; 4 Instituto Oswaldo Cruz, Fundação Oswaldo Cruz, Rio de Janeiro, 21040-900 Brazil; 5 Faculty of Sciences, University of South Bohemia, 370 05 České Budějovice, Czechia; 6 Martsinovsky Institute of Medical Parasitology, Sechenov University, Moscow, 119435 Russia

**Keywords:** *Herpetomonas muscarum ingenoplastis*, Cell cycle, Flagella connector, *Trypanosoma brucei*

## Abstract

**Background:**

The family Trypanosomatidae encompasses parasitic flagellates, some of which cause serious vector-transmitted diseases of humans and domestic animals. However, insect-restricted parasites represent the ancestral and most diverse group within the family. They display a range of unusual features and their study can provide insights into the biology of human pathogens. Here we describe *Vickermania*, a new genus of fly midgut-dwelling parasites that bear two flagella in contrast to other trypanosomatids, which are unambiguously uniflagellate.

**Results:**

*Vickermania* has an odd cell cycle, in which shortly after the division the uniflagellate cell starts growing a new flagellum attached to the old one and preserves their contact until the late cytokinesis. The flagella connect to each other throughout their whole length and carry a peculiar seizing structure with a paddle-like apex and two lateral extensions at their tip. In contrast to typical trypanosomatids, which attach to the insect host’s intestinal wall, *Vickermania* is separated from it by a continuous peritrophic membrane and resides freely in the fly midgut lumen.

**Conclusions:**

We propose that *Vickermania* developed a survival strategy that relies on constant movement preventing discharge from the host gut due to intestinal peristalsis. Since these parasites cannot attach to the midgut wall, they were forced to shorten the period of impaired motility when two separate flagella in dividing cells interfere with each other. The connection between the flagella ensures their coordinate movement until the separation of the daughter cells. We propose that *Trypanosoma brucei*, a severe human pathogen, during its development in the tsetse fly midgut faces the same conditions and follows the same strategy as *Vickermania* by employing an analogous adaptation, the flagellar connector.

## Background

Flagellates of the family Trypanosomatidae (Euglenozoa: Kinetoplastea) are obligatory parasites inhabiting vertebrates, arthropods, leeches, and plants [1, 2]. Since numerous dixenous trypanosomatids, i.e., those alternating between vertebrate and invertebrate hosts, cause diseases in humans and domestic animals, they were given most attention [3–5]. However, the insect-restricted monoxenous trypanosomatids are ancestral and more diverse, with some of their representatives having unusual features, such as an idiosyncratic genetic code [6], bacterial endosymbionts [7–9], and novel viruses [10, 11].

For a long time, the classification of monoxenous trypanosomatids was based solely on morphology, with each genus characterized by a predominant and/or unique morphotype [12, 13]. Because of the deficit of morphological features, species delimitation was even more precarious. The situation was ameliorated by sequence-based approaches that provided a significantly higher resolution [14] and allowed testing old morphological descriptions in the molecular phylogenetic framework [15].

*Herpetomonas muscarum* is the first named monoxenous trypanosomatid, originally reported in 1856 from the housefly *Musca domestica* in the USA [16]. Later, flagellates from about 30 dipteran species of eight families, sampled around the world, were classified as *H. muscarum* [1, 17]. The first detailed characterization of this species pointed to the presence of two united flagella [18], a peculiar feature proposed to be diagnostic for the genus *Herpetomonas*. However, many authors criticized this idea, arguing that biflagellate cells represented nothing but dividing forms, and, therefore, synonymized *Herpetomonas* with *Leptomonas* and even *Leishmania* [19–23]. Afterwards, the proposal to distinguish *Herpetomonas* from *Leptomonas* based on the presence of a particular morphotype, now termed opisthomastigote [12], became widely adopted [24, 25].

The biflagellate cells were forgotten for over 50 years and brought to light again only in 1971, when two cultures of morphologically distinct trypanosomatids were isolated from flies. These were described as two subspecies of *Herpetomonas muscarum*: *H. m. muscarum* with predominantly uniflagellate cells possessing a narrow rod-shaped kinetoplast and *H. m. ingenoplastis* with mainly biflagellate cells bearing a larger teardrop-shaped kinetoplast [26]. Later, comparative studies of these two subspecies revealed that they also substantially differ in proliferation rates, metabolism, and sizes of the kinetoplast DNA (kDNA) circles [27–31].

However, little attention was paid to the phenomenon of double flagella, the most striking feature of *H. m. ingenoplastis.* All other known trypanosomatids are uniflagellate and possess second flagellum only for a short period, during cell division [32, 33]. Outside trypanosomatids, all motile kinetoplastids have two flagella, but those are heterodynamic: one is oriented anteriorly and the other posteriorly [34]. In this study, we have performed a morphological and molecular re-description of *H. m. ingenoplastis* and a newly isolated related species, and studied their development both in culture and in experimentally infected flies. Our results demonstrate that both species, now assigned to a new trypanosomatid genus *Vickermania*, have a very unusual kinetoplast and suggest that they became biflagellate to adapt to life in the midgut of flies.

## Results

### Light microscopy and cultivation

**Fig. 1:**
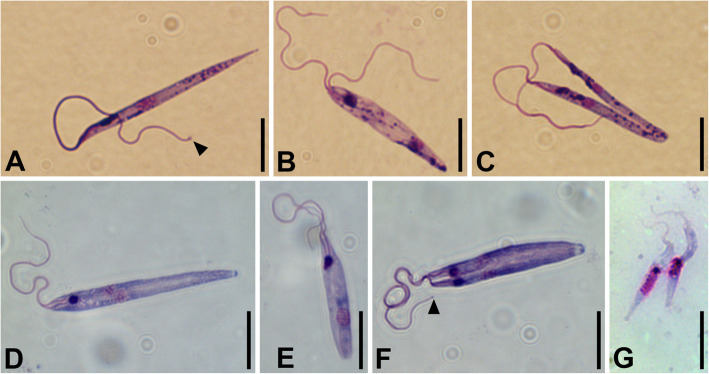
Cells on Giemsa-stained smears. **a**–**c** Culture CP021. **d**–**f** Culture S13. **g** Epimastigotes in S13-gut sample. Arrowhead points to the terminal thickening of the flagellum. Scale bars are 10 μm

Light microscopic examination of the axenic cultures CP021 (replica of *Herpetomonas muscarum ingenoplastis* type culture) and S13 (newly isolated from an ensign fly) as well as the xenic culture Trypanomatidae sp. F72 (newly isolated from a green bottle fly) revealed the presence of a single morphotype—promastigotes (Fig. 1, Additional file 1: Fig. S1). In all studied strains, their size was similar (Additional file 2: Table S1) and did not change during cultivation, although the maximal lengths of cell and flagella in S13 exceeded those in others. In all cultures, some promastigotes appeared uniflagellate, although their flagellum was often conspicuously thicker in the proximal part (Fig. 1a); others had two completely separate flagella (Fig. 1b) with a noticeable thickening at their tip (Fig. 1a, f). In addition, there were many intermediate variants with flagella being separated only until a certain point (Fig. 1c–f), sometimes only inside the flagellar pocket.

The examination of the smear made from the naturally infected fly intestine, which served as a source of the S13 culture (sample S13-gut), revealed that the majority of cells were strikingly different from those in the culture. They had epimastigote morphology and a conspicuously shorter free flagellum, no longer than half of the cell body (Fig. 1g).

The cultures CP021 and S13 showed similar growth dynamics with approximately the same maximum density (6–9 × 10^6^ cells/ml) reached on day 5, followed by a rapid decline (Additional file 3: Fig. S2). The proportion of unambiguously biflagellate cells (i.e., those with well-separated flagella), as estimated for CP021, was minimal during the intense growth phase (< 5%) and increased to 17–35% in old cultures (day 9).

### Molecular phylogenetic analyses

The comparison of the newly obtained 18S rRNA gene sequences revealed that they belong to three separate species. The first of them included indistinguishable CP021 and F72, the second—S13 (97.13% identical to the first), while the third one—from the naturally infected fly intestine (sample S13-gut)—shared 94.05% and 93.13% identity with the above two species, respectively.

**Fig. 2:**
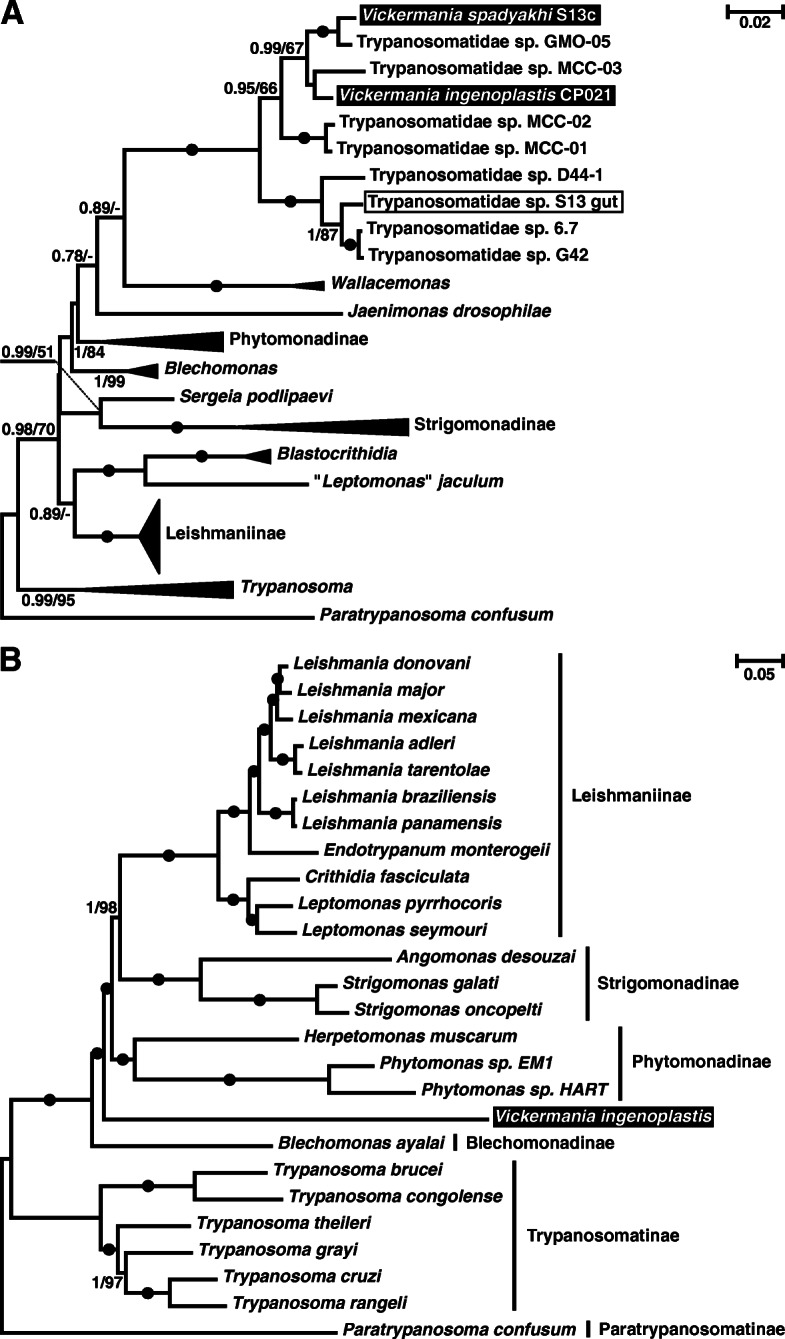
Maximum likelihood phylogenetic trees of trypanosomatids. **a** Tree based on 18S rRNA gene sequences. **b** Tree based on 160 concatenated protein sequences. Numbers at nodes indicate posterior probability and bootstrap percentage, respectively. Values below 0.5 or 50% are replaced with dashes or omitted, absolute (1/100) supports are shown as black circles. The scale bar denotes number of substitutions per site. All clades representing described subfamilies or genera are collapsed in the 18S rRNA gene-based tree. The two species of biflagellate trypanosomatids studied here are highlighted; the sample S13-gut from the naturally infected *Nemopoda nitidula* is boxed

On the inferred 18S rRNA gene tree (Fig. 2a), all three species fell into a clade that had maximal support on both ML and Bayesian trees and consisted of two subclades. The first one includes S13-gut sample along with the isolates 6.7, G42 and D44-1 from *Drosophila ananassae*, the assassin bug *Nagusta* cf. *punctaticollis*, and feces of a great ape, respectively [35–37]. The second subclade, with a high posterior probability and a moderate bootstrap support, contained CP021 and S13, as well as the isolates MCC-01 from an unidentified fly, MCC-02 and MCC-03 from *Drosophila* sp., and GMO-05 from *Musca* sp. [38, 39]. Although both applied phylogenetic methods placed *Wallacemonas* as a sister group to the clade under discussion, and *Jaenimonas drosophilae* as the next closest neighbor, these relationships were poorly supported. The removal of either of these two genera from the dataset resulted in a slight increase of the bootstrap supports (to 55–61%) and more significant rise of the posterior probabilities (to 0.97–0.99), supporting the association of the clade under question with the remaining taxon (data not shown).

The only other well-sampled marker that has been widely used for inference of trypanosomatid phylogeny is the glycosomal glyceraldehyde-3-phosphate dehydrogenase (gGAPDH) gene [40]. However, it was shown to produce artifacts, due to the compositional bias in nucleotide sequences [8, 9, 41, 42]. By using the amino acid sequences of gGAPDH, we attempted to benefit from the large available dataset, while decreasing the risk of artifacts (Additional file 4: Fig. S3). This analysis recovered genus-level clades with moderate-to-maximal supports, but virtually failed at a higher level: Strigomonadinae appeared paraphyletic, while Phytomonadinae, though monophyletic by ML, had a very low bootstrap support. Nevertheless, the gGAPDH sequences of CP021 and S13 formed a clade with maximal support, which also includes an undescribed parasite of *Drosophila obscura* and *D. tristis* [43]. Thus, CP021 and S13 belong to a lineage without a taxonomic name. Based on their separate phylogenetic position and distinct morphology, we propose to accommodate them in a newly erected genus *Vickermania* (see the “Taxonomic summary” section for details).

Given the availability of the recently published genomic sequence of the isolate CP021 [44], we decided to take advantage of the high-resolution power inherent to phylogenomic analysis. The ML and Bayesian trees inferred using concatenated sequences of 160 proteins invariantly placed *Vickermania* as an independent lineage branching off the trypanosomatid stem right after Blechomonadinae and thereby being sister to the assemblage of the subfamilies Leishmaniinae, Strigomonadinae, and Phytomonadinae (Fig. 2b). Regrettably, the lack of genomic data for *Wallacemonas* and *Jaenimonas* did not allow testing whether any of these two genera is related to *Vickermania* as suggested by the analysis of the 18S rRNA gene.

### Transmission electron microscopy

**Fig. 3:**
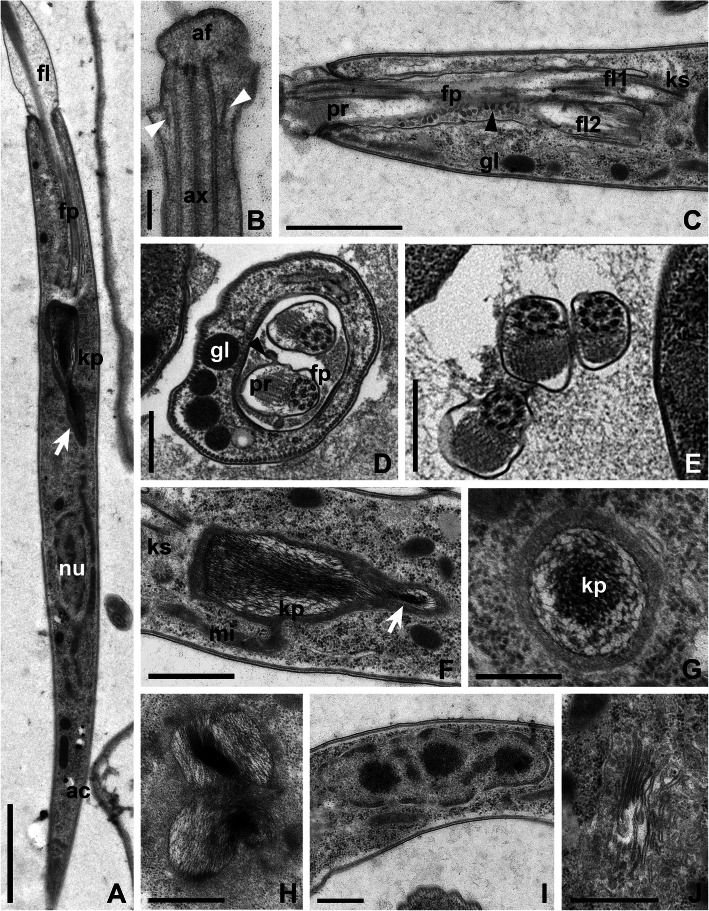
Ultrastructure of the strain CP021*.* **a** General view of the anterior part of the cell. **b** Flagellar tip. **c** Longitudinal section through flagellar pocket. **d** Transverse section through a flagellar pocket with two flagella. **e** Four kinetosomes (indicated by numbers). **f, g** longitudinal and transverse sections of the kinetoplast. **h** Dividing kinetoplast. **i** Golgi apparatus. **j** Interphase nucleus of irregular shape. Black arrowhead shows vesicles detaching from the flagellar membrane, white arrowheads—oblique fibrils between the axoneme and plasmalemma, white arrows—kinetoplast appendage containing kDNA fibrils, asterisk—microtubules supporting the flagellar pocket wall. Abbreviations: ac, acidocalcisomes; af, apex of the flagellar tip; ag, Golgi apparatus; ax, axoneme; fl, flagella; fp, flagellar pocket; gl, glycosomes; kp, kinetoplast; ks, kinetosomes; nu, nucleus; pr, paraxial rod. Scale bars: **a** 2 μm, **b** 0.25 μm, **c**, **e** 1 μm, **d**, **f**–**j** 0.5 μm

**Fig. 4:**
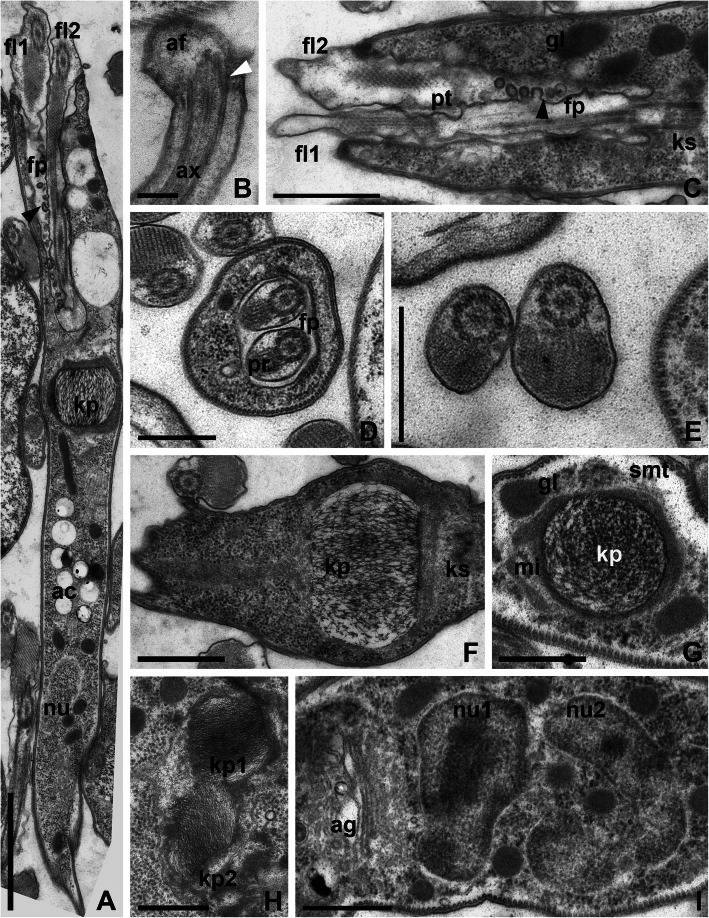
Ultrastructure of the strain S13*.* **a** General view of the anterior part of the cell. **b** Flagellar tip. **c** Longitudinal section through flagellar pocket. **d** Transverse section through a flagellar pocket with two flagella. **e** Four kinetosomes (indicated by numbers). **f**, **g** Longitudinal and transverse sections of the kinetoplast. **h** Dividing kinetoplast. **i** Golgi apparatus and two interphase nuclei of irregular shape. All symbols and abbreviations are the same as in Fig. 3. Scale bars: **a** 2 μm, **b** 0.25 μm, **c***,* e 1 μm, **d**, **f**–**i** 0.5 μm

At the ultrastructural level, CP021 and S13 are generally similar to most trypanosomatids studied to date. Flagella with basal bodies, kinetoplast, nucleus, and Golgi apparatus were situated in the anterior part of the cell (Figs. 3a and 4a). The flagellar pocket was 3–4 μm deep and 0.9–1.2 μm in diameter. Surprisingly, the membrane of flagella inside the pocket was plicate and appeared to be involved in active exocytosis (Figs. 3c and 4c). The paraflagellar rod was perceptible starting from the middle part of the flagellar pocket (Figs. 3d and 4a, c, d), upon the exit of which the flagella became thicker owing to the increase of the flagellar matrix volume (Figs. 3a, c and 4a, c). In cross sections, the flagella outside the pocket were often seen close to each other or even adjacent, yet no specific structures could be observed at the site of the contact (Figs. 3e and 4e). In both examined species, the flagellar tip had a peculiar organization: a knob-like apex was situated distally to the ending of the axonemal microtubules, while proximally to it small lateral extensions were observed. These extensions were internally reinforced by oblique fibrils running from the peripheral microtubules of the axoneme to the flagellar membrane at an angle of 30–45° (Figs. 3b and 4b).

In both species, the cytoplasm contained multiple glycosomes, acidocalcisomes and a reticulated mitochondrion with rare cristae (Figs. 3f–h and 4a, f–h). The kDNA arrangement in these flagellates was unusual, with prominent differences between the two studied strains. In CP021, the kDNA mass had a dense longitudinally oriented central kernel and looser periphery (Fig. 3f, g). On average, it measured 2.1 μm in length and 0.6 μm in diameter (in its widest part). This mass possessed a posterior appendage that gave it the shape of a drop. In S13, the kDNA mass was barrel-shaped (Fig. 4f) with the height and the diameter at both ends being roughly equal (0.6–0.8 μm) and the widest central part measuring ~ 1 μm in diameter. The kDNA in this trypanosomatid was distributed rather uniformly and in general looked looser as compared to CP021 (Fig. 4f, g). In both species, the kDNA divided along the longitudinal axis (Figs. 3h and 4h), and in CP021, the central kernel became heavily condensed. Usually stretched along the longitudinal cell axis, the nuclei were mostly oval in CP021 and irregularly shaped in S13 (Figs. 3i and 4i). The Golgi apparatus composed of 8 to 10 cisternae was located between the kinetoplast and the nucleus (Figs. 3j and 4i).

### Scanning electron microscopy

**Fig. 5.**
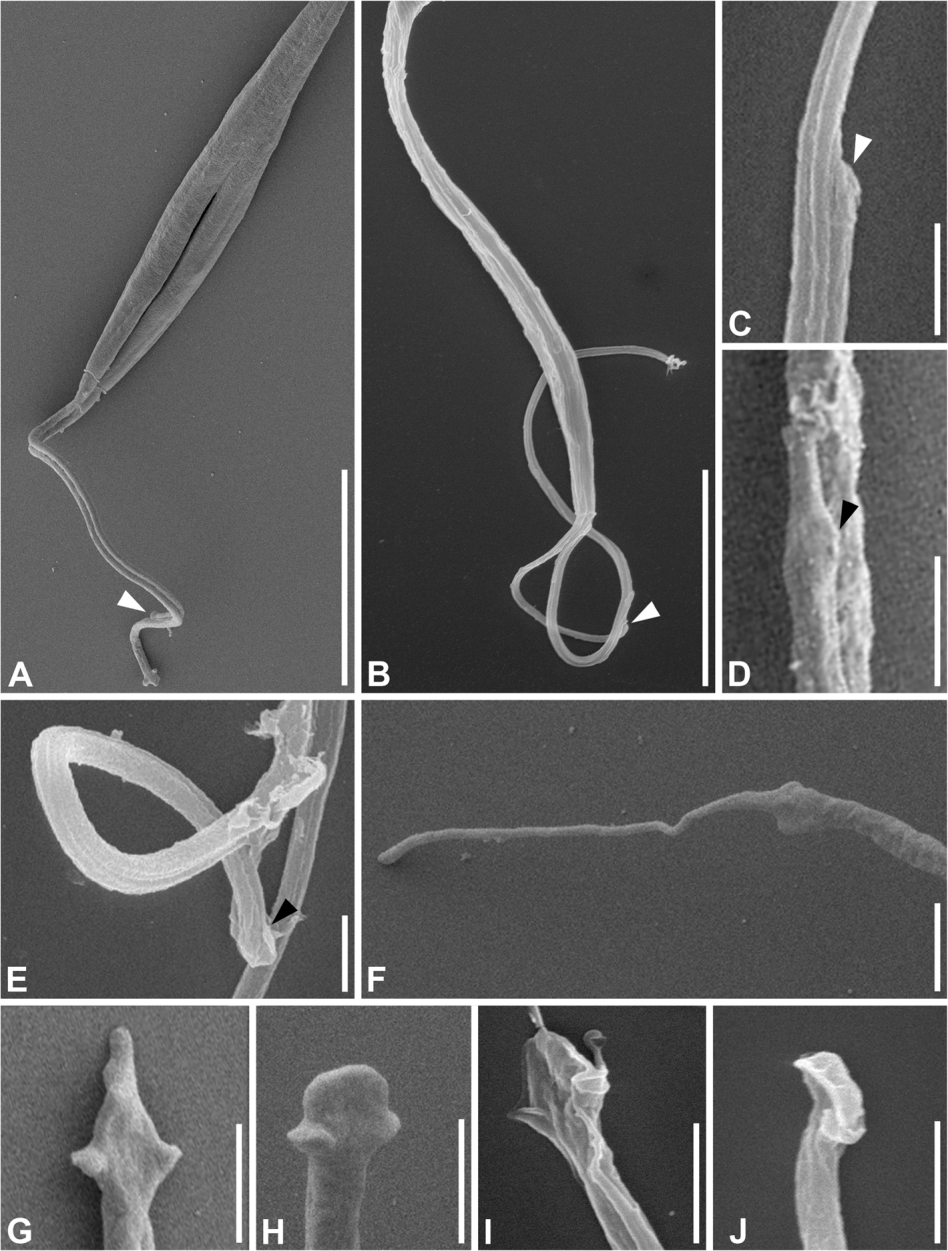
Scanning electron microscopy of the strains CP021 and S13. **a** Joint flagella during cytotomy in CP021. **b** Non-dividing cell of S13: the second flagellum is attached only by its tip. **c** Lateral view of the tip of the second flagellum tightly connected to the first one in S13. **d** Top view of the second flagellum’s tip in CP021; note the elongated distal part and lateral extensions. **e** Detaching flagellar tip in S13. **f–j** Free flagellar tips in CP021 (**f–h**) and S13 (**i, j**). White arrowhead—tip of the second flagellum; black arrowhead—lateral extension of the flagellar tip. Scale bars: **a**, **b** 10 μm, **c**–**j** 1 μm

Examination of the trypanosomatids under scanning electron microscope clarified any uncertainty regarding the number of flagella. In both species, the two flagella were tightly connected even in the cells starting cytokinesis (Fig. 5a). The shorter flagellum often remained connected by its tip to the longer one even if the flagella were partially separated (Fig. 5b). In the side view, the attached tips appeared tapered (Fig. 5c), while different shapes could be seen in the top view. In CP021, the flagellar tip was reminiscent of a crocodile head showing lateral extensions and an elongated central part, while in S13 it resembled a paddle with lateral extensions and a blunt apex (Fig. 5d, e). Free tips typically demonstrated more prominent (as compared to the attached ones) lateral extensions and had diverse apex shapes that were either filiform (Fig. 5f), triangular (Fig. 5g), or paddle-like (Fig. 5h) in CP021, and mostly paddle-like in S13 (Fig. 5i, j).

### Experimental infections

Thirty minutes after the flies ingested the liquid containing trypanosomatids, the flagellates localized mainly in the crop, with one or two detected in the anterior midgut (data not shown). Starting from the first day post-infection, the promastigotes, the only observable morphotype, were present in all sections of the midgut except the proventriculus, with a few cells occasionally present in the hindgut and feces. Importantly, all flagellates were detected only in the endoperitrophic space and were never attached to any substrate (Additional file 5: Fig. S4).

The infection of *Calliphora vicina* by the CP021 strain was transitory, as no flagellates were observed 5 days post infection (Table 1). The S13 strain was able to inhabit this host for at least 15 days, but only about half of the flies remained infected, and the horizontal transmission experiments were not successful. Both trypanosomatids were able to survive in *Lucilia sericata* until the end of the experiments and single-infection longevity experiments showed high prevalence (87 and 100% for CP021 and S13, respectively), yet only S13 could be horizontally transmitted (Table 1). The infections of *L. sericata* with small numbers of flagellates (~ 25) showed effective propagation of S13 (184–220 and 2260–2530 cells counted 3 and 5 days post-infection, respectively), while the numbers of CP021 gradually decreased (3–12 and 0–2 cells for days 3 and 5, respectively). Hence, *L. sericata* was selected as a model host for a subsequent study of the cell cycle in vivo.

**Table 1:** Prevalence (infected/dissected ratio) of trypanosomatids in experimentally infected flies

Host/parasite combination	Day 1	Day 3	Day 5	Day 15	Isolation (day 20)	Horizontal transmission
*Calliphora vicina*/CP021	3/3	6/6	0/10	n/a	n/a	n/a
*Calliphora vicina*/S13	3/3	6/6	4/10	4/9	n/a	0/10
*Lucilia sericata*/CP021	5/5	5/8	6/12	3/7	20/23	0/20
*Lucilia sericata*/S13	5/5	6/6	6/6	10/10	20/20	6/21

### Cell cycle

**Fig. 6.**
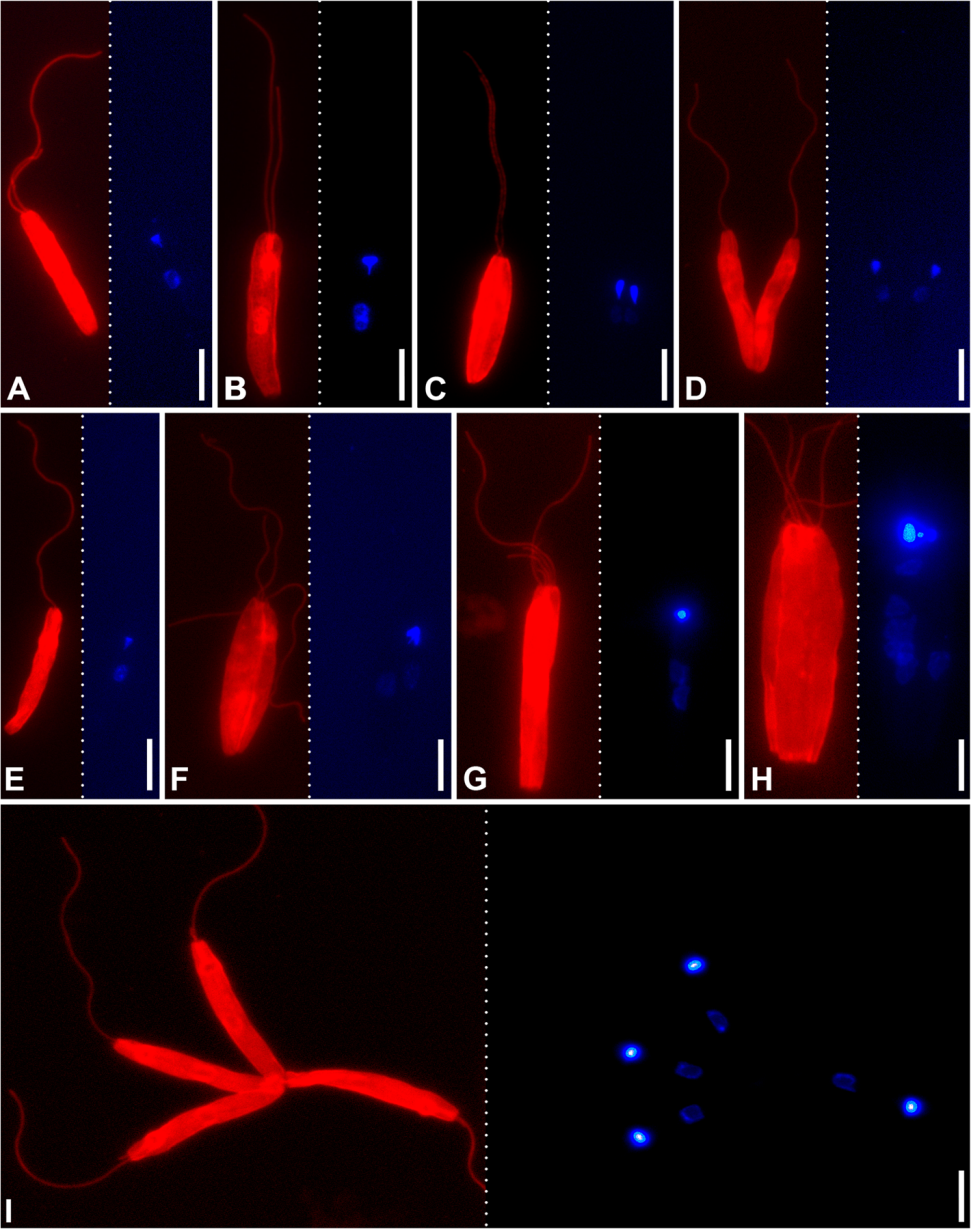
Various stages of the cell cycle and division anomalies. Fluorescent visualization using anti-α-tubulin antibodies and DAPI are shown on the left and right subpanels, respectively. **a**–**f** CP021 strain. **g**–**i** S13 strain

In order to investigate the dynamics of the flagella during the cell cycle, the axonemes and DNA in nuclei and kinetoplasts were visualized using antibodies against alpha-tubulin and DAPI or SYTO24 dyes, respectively. The studies were performed using cells of both species in cultures and from the intestine of experimentally infected *L. sericata* flies (Table 2). In all cases, the most abundant (> 57%) cell type with two flagella, one nucleus and one kinetoplast (Fig. 6a), cells undergoing regular division (Fig. 6b–d), and those with a single nucleus, kinetoplast and flagellum (Fig. 6e), constituted overwhelming majority (~ 89% in cultures and ~ 98–99% in the gut) (Table 2). Importantly, in both species, cells with more than two flagella were observed only in the culture and appeared to be associated with division anomalies (Fig. 6f–i). Hence, the normal cell cycle includes alternation of the biflagellate and uniflagellate stages, with the latter being the product of a recent division of the former. Of note, CP021 demonstrated dramatic depletion of dividing cells in the gut as compared to the culture, while in S13 the proportions of such cells did not differ between these conditions (Table 2).

**Table 2:** Proportions of different types of cells in cultures and experimentally infected flies

	Number of flagella/nuclei/kinetoplasts					Sample size
	2/1/1	1/1/1	2/2/2, 2/1/2, 2/2/1	> 2 flagella	Others	
CP021						
culture	60.9%	16.3%	12.0%	5.8%	5.1%	276
gut	80.8%	16.4%	0.7%	0.0%	2.1%	287
*p* value*	< 0.001	0.97	< 0.001	< 0.001	0.055	
S13						
culture	57.5%	23.6%	7.5%	4.7%	6.6%	212
gut	64.3%	28.4%	6.2%	0.3%	0.5%	370
*p* value*	0.1	0.21	0.55	< 0.001	< 0.001	

*Calculated using *χ*^2^ test

The second flagellum appears to grow continuously. Even in the dividing cells, it is always shorter and its average ratio in length to the first one is roughly equal in both species, 0.81 ± 0.09 (0.56–0.98) in CP021 and 0.80 ± 0.10 (0.61–0.99) in S13. This suggests that the flagella continue growing even after the completion of cell division.

### Analysis of cell motility

**Fig. 7:**
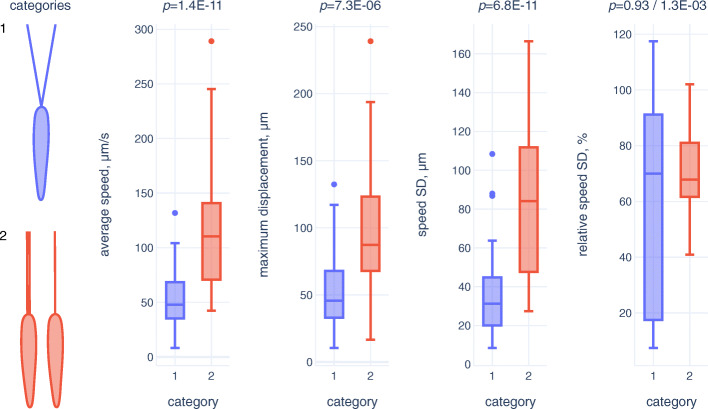
Comparative analysis of cell motility. Two categories of cells (with two separate flagella and with a single or two joint flagella) are compared. The boxes and whiskers correspond to interquartile and non-outlier ranges, respectively. Mann-Whitney *U* test only or both Mann-Whitney *U* test and Wald-Wolfowitz runs test *p* values are shown above the graphs

In order to test the hypothesis that the flagella separation results in lowered motility, we performed a comparative analysis of in vitro swimming behavior for two cell categories: (1) with two separate flagella and (2) with a single or two joint flagella. The cells with separate flagella showed significantly lower values of average speed, maximum displacement and absolute speed changes (estimated as standard deviation [SD] of speed) being a proxy for agility (Fig. 7; Additional file 6: Table S2). The differences were so pronounced that interquartile ranges for all these parameters in the two cell categories did not overlap (Fig. 7). Expectedly, average speed and maximum displacement showed statistically significant correlation in both cell categories, while the intuitive correlation of the average speed and absolute speed changes (faster cells lose more speed when changing direction and accelerate more on nearly straight trajectory sections) could be observed only for the category 2 (Additional file 7: Table S3). At the same time, only the category 1 displayed a moderate correlation between the absolute and relative speed changes (relative SD of speed). This agreed with significantly greater (as judged by Wald–Wolfowitz runs test) variation of relative speed changes in the category 1, as compared to its counterpart, while the differences in median values were negligible (Fig. 7; Additional file 6: Table S2). This can be interpreted as more uniform speed patterns in the category 2, representing predominant (~normal) cells, while disturbances of motility caused by flagella separation lead to increase in the spectrum of aberrations. Moreover, inspection of the cell trajectories suggests that faster cells of the category 1 make less turns than those in the category 2, i.e., they are less agile (Additional files 8, 9: Suppl. Fig. S5, S6).

### Taxonomic summary

Since this publication contains nomenclatural changes, it has been registered in Zoobank: urn:lsid:zoobank.org:pub:73A2FCA2-F35B-4050-8EE7-DCAFCAEBD770.

Class: Kinetoplastea Honigberg, 1963

Order: Trypanosomatida Kent, 1880

Family: Trypanosomatidae Doflein, 1901

Genus: *Vickermania* Kostygov et Yurchenko, 2020 gen. nov.

Zoobank LSID: urn:lsid:zoobank.org:act:F31B2BA7-A896-49B8-A5CF-4A6F3BD4FB6F

Diagnosis: promastigotes with two anteriorly oriented flagella of unequal length, typically attached to each other, separated only during cell division; uniflagellate cells appear only shortly after division, as the second flagellum starts growing soon after it; flagellar tips have rounded or elongated apex and lateral extensions; large and loosely arranged kDNA.

Name-bearing type: *Herpetomonas muscarum ingenoplastis* Rogers and Wallace, 1971 now classified as *Vickermania ingenoplastis*

Etymology: The generic name honors Prof. Keith Vickerman, an outstanding British protistologist, who focused on studies of various parasitic protists, mainly trypanosomatids.

*Vickermania ingenoplastis* (Rogers et Wallace, 1971) comb. nov., stat. nov.

Species diagnosis and description: up to 80% cells in culture are biflagellate; cells are 27.3–40.7 μm long and 2.3–3.5 μm wide; flagellum is 28.8–44.5 μm long; kDNA mass is about 2.1 μm long, 0.6 μm in diameter and has grape seed shape; it has loose periphery, and dense kernel.

Type host: *Phormia regina* (Diptera: Calliphoridae)

Localization: midgut

Type locality: Minneapolis–Saint Paul, Minnesota, USA.

Type material: the culture ATCC30259.

Gene sequences: MT241904 (18S rRNA gene), and MT248932 (gGAPDH gene), KX901631 (draft genome).

Comments: (i) opisthomastigotes and short promastigotes reported in the original description [26] were not observed in our study neither in the culture nor during the development in the host gut and most likely had represented an admixture by some *Herpetomonas* sp., which was lost as a result of long-term cultivation; (ii) the culture F72 isolated from *Lucilia sericata* (Calliphoridae) in Oksochi, Novgorod Oblast, Russia (58° 39′ N, 32° 7′ E), belongs to the same species according to the 18S rRNA gene sequences.

*Vickermania spadyakhi* Ganyukova et Kostygov sp. nov.

Zoobank ID: urn:lsid:zoobank.org:act:D68B3910-9D91-402B-8FEC-2CF9AF1506AF.

Species diagnosis and description: up to 60% cells in culture are biflagellate; cells are 22.5–75.9 μm long and 1.8–4.7 μm wide; flagellum is 25.6–64.0 μm long; barrel-shaped kDNA mass with diameter of 0.6–0.8 μm at both ends and 1 μm in center.

Type host: *Nemopoda nitidula* (Diptera: Sepsidae)

Localization: midgut

Type locality: Sob’ railway station, Yamalo-Nenets Autonomous Okrug, Russia (67° 06′ N, 65° 71′ E)

Type material: the culture S13, deposited in the research collection of Parasitic Protists of the Zoological institute RAS (St. Petersburg, Russia).

Etymology: the specific epithet has Russian origin and means “from near Padyakha”, referring to the local name of the river Sob’, close to which the species was collected.

Gene sequences: MT241903 (18S rRNA gene), and MT248930 (gGAPDH gene).

Comment: the type culture was established from an insect with a mixed infection, including morphologically dissimilar epimastigote-type cells with a distinct phylogenetic position.

## Discussion

Since the description of biflagellate trypanosomatids more than a century ago by Stanislav Provázek, the discoverer of epidemic typhus [18], their very existence was repeatedly questioned, as it conflicted with the idea that a single flagellum is a distinctive feature of the family Trypanosomatidae, clearly separating them from other kinetoplastids, which are biflagellate or aflagellate [45]. However, the records of this peculiar morphotype from a range of fly hosts in different geographic areas [18, 26, 27, 46], and the fact that both flagella are usually glued together, prove that this is not an abnormality but a stable morphological adaptation.

Our study confirmed that *Vickermania* is not truly biflagellate in the sense that it lacks a tetraflagellate stage prior to the cell division, but instead becomes transiently uniflagellate right after its completion. However, these trypanosomatids bear two flagella throughout most of their cell cycle and, in this respect, shall be considered biflagellate. In order to achieve this, *Vickermania* had to disconnect the well-established ancestral coordination of the cell cycle, and this results in a relatively high percentage of anomalous forms associated with various cell division disturbances.

Why did then *Vickermania* become biflagellate? Taking into account that the main function of the flagellum is locomotion [47], this is likely to be related to cell motility. Indeed, it seems to be important for cell survival as judged by long flagella strengthened by a thick paraflagellar rod. Although it is tempting to speculate that two flagella ensure more efficient movement, this seems unlikely. Firstly, the uniflagellate cells constitute a significant proportion and are not depleted in the gut, where the conditions are more restrictive than in the culture. Secondly, in many biflagellate cells, the second flagellum is rather short and can hardly enhance motility. While in a typical trypanosomatid the second flagellum appears only at the end of the cell cycle and is not connected to the old one [33], *Vickermania* preserves a connection between the two flagella for as long as possible. This allows them acting as a single entity, not interfering with each other due to possible uncoordinated beating. Our analysis of motility confirmed that the cells with a single or two joint flagella are significantly faster and more agile as compared to those with two separate flagella. The observed low frequency of cells with separate flagella in the log-phase and its increase only in degrading post-stationary cultures further supports this assumption. In order to build the new flagellum in time, the trypanosomatid starts growing it in the very beginning of the cell cycle. The peculiar “seizing” structure of the flagellar tips likely mediates a firm attachment and a proper orientation of the growing flagellum.

Why may motility be of higher importance to *Vickermania* than to other trypanosomatids? As highly diverse and successful parasites, trypanosomatids evolved a range of strategies allowing them to colonize a wide range of hosts [48]. The evolutionarily primary and still most frequent localization of these flagellates within the insect host is the intestine [49], which allows direct access to the digested food, while both the entry and exit from this organ are straightforward. However, the parasites have to evolve adaptations preventing their premature discharge, typically by attaching to the intestinal wall via various modifications of the flagellum [50–58]. Although *Vickermania* thrives in the midgut, it cannot implement attachment, since in its fly host the midgut wall is separated from the lumen by a semipermeable continuous structure known as the peritrophic membrane [59]. It is produced by secretory cells at the midgut entrance, continuously grows in the posterior direction, becomes partially destroyed in the hindgut, and is eventually discharged with feces [60]. Attachment to this structure would lead to a rapid removal of flagellates from the gut. Some intestinal protists such as human-infecting *Leishmania* spp. can penetrate this barrier during their development in sandflies [61]. However, the peritrophic membrane of sandflies is short-lived and starts disintegrating at about the third day post blood meal (long before defecation), significantly facilitating the escape of parasites [62]. A coexistence with the permanent peritrophic membrane apparently forced *Vickermania* to rely only on an active movement against the flow created by intestinal peristalsis. In such harsh conditions, dividing cells are vulnerable due to their decreased motility. Indeed, in the strain CP021, which showed lower infection efficiency in the model fly species (probably because it had been already cultivated for over 50 years), the proportion of dividing cells displayed a dramatic drop during development in the gut. Since bearing two separate flagella in such conditions can make cells vulnerable, *Vickermania* has shortened this period. Regrettably, very little is known about the development of other monoxenous trypanosomatids inhabiting the fly midgut [49], and therefore, it is not possible to compare the efficiency of this and alternative strategies.

The procyclic trypomastigotes of *Trypanosoma brucei* live in the endoperitrophic space of the tsetse fly midgut, where they also have no chance to attach to the intestinal wall. Interestingly, this developmental stage demonstrates a surprising analogy to *Vickermania*, as its newly growing flagellum is also connected by the tip to the old one [63]. However, the mechanism utilized by *T. brucei* is different: it developed for this purpose a specialized structure called the flagella connector, which is absent from all other developmental stages [64]. This structure was proposed to mediate inheritance of a cellular pattern [65], but in the light of our study can be seen from a different perspective. The alternative reason may be retaining efficient motility by preserving coordination between the two flagella, a critical requirement for survival in the fly midgut. Indeed, the principle of the Red Queen “it takes all the running you can do, to keep in the same place”, seems to be particularly fitting here. However, *T. brucei* eventually migrates from the intestine into the salivary glands, where it becomes attached to the epithelium, while *Vickermania* is doomed to permanent movement.

## Conclusions

The new genus *Vickermania* represents overlooked, yet widely distributed trypanosomatids that adapted to the life in the fly midgut, where the default trypanosomatid strategy, namely the attachment to the intestinal epithelium, is impossible. Instead, they rely on constant movement, preventing discharge from the host gut due to intestinal peristalsis. Therefore, these parasites shortened the period of the cell cycle, when they bear two separate flagella interfering with each other and thus become vulnerable. This is achieved by disconnecting the flagella duplication from cell division and by developing a mechanism to join the new growing flagellum with the old one. Our findings also shed light on the biology of the deadly human pathogen *Trypanosoma brucei*. During development in the tsetse fly midgut, it likely faces the same conditions and follows the same strategy as *Vickermania*. The flagella connector, unique for the gut stage of *T. brucei*, and the peculiar seizing structure at the tip of the *Vickermania* flagellum developed independently, but play the same role ensuring efficient motility in dividing cells. These remarkably analogous adaptations demonstrate the plasticity of one of the main eukaryotic cellular structures, the flagellum.

## Methods

### Trypanosomatid strains: origin and cultivation

The CP021 culture of *H. m. ingenoplastis* was obtained from the Fiocruz Protist Culture Collection. It is a replicate of the type culture ATCC30259, isolated from *Phormia regina* (Calliphoridae) [26]. The culture S13 originated from the midgut of the fly *Nemopoda nitidula* (Sepsidae) collected in July 2016 near Sob’ railway station (67° 06′ N, 65° 71′ E), Yamalo-Nenets Autonomous Okrug, Russia, as described elsewhere [66]. The intestinal fragments of the infected fly were also smeared for microscopy and used for DNA isolation (sample S13-gut). The culture F72 was isolated from the midgut of *Lucilia sericata* (Calliphoridae) captured in May 2019 in the village Oksochi (58° 39′ N, 32° 47′ E), Novgorod Oblast, Russia. To date it could not be purified from the accompanying fungal contaminants.

All trypanosomatid cultures were maintained at 23 °C in Schneider’s Drosophila medium (Sigma-Aldrich, St. Louis, USA) supplemented with 10% fetal bovine serum (FBS) (Thermo Fisher Scientific, Waltham, USA), 500 μg/ml of streptomycin and 500 Units/ml of penicillin (Sigma-Aldrich), and passaged weekly. In order to study growth dynamics, the cultures CP021 and S13 were seeded in hexaplicates with a starting concentration of 40,000 cells/ml and subsequent counting with hemocytometer at days 1, 2, 3, 5, 7, 9, and 11.

### Experimental infections

Experimental infections were performed using laboratory cultures of the flies *Calliphora vicina* and *Lucilia sericata* (both Calliphoridae), maintained at the Zoological Institute, St. Petersburg, since 2010. The insects were cultivated at 25 °C, with larvae being fed pork liver, while adults received sugar cubes and clean water. Two days after hatching from pupae, 50% of individuals were used for experiments, while the remaining half underwent control dissection and were always trypanosomatid-negative. Before the experiments, the flies were kept without water for 24 h.

The thirsty flies were given access to a bowl containing flagellates in cultural medium, which was replaced after 24 h with clean water. From that moment, the time of infection was counted and the dissections were performed on days 1, 3, 5, and 15. The possibility of intraspecific horizontal transmission was tested by placing 5 uninfected flies with 5 recently (1–2 days) infected ones. Ten days later, all insects were dissected.

In order to assess the duration of single infections, individual infected flies were isolated in plastic tubes of a diameter that prevented them from turning around and, thus, being re-infected with their own feces. The tube opening was closed with a fine-meshed net, through which the fly had access to a piece of cotton wool soaked in 17% sugar syrup. At the posterior end, the tube contained a piece of dry cotton wool absorbing excreta (Additional file 10: Fig. S7).

For determining the efficiency, localization, and propagation of an infection initiated with a small parasite dose, flies were given a 5-μl drop of the culture medium containing ~ 25 trypanosomatid cells, and 5 individuals were dissected at 30 min, 3 and 5 days post-infection.

The work with flies did not require specific permissions, since they do not belong to an endangered or protected species.

### Microscopy

The trypanosomatid cells on Giemsa-stained smears were photographed using DM 2500 Leica microscope and measured with Fiji software [67]. For fluorescent microscopy, cells from either 3-day-old culture or the intestinal contents of flies infected 3 days before were centrifuged, fixed with 4% paraformaldehyde in phosphate-buffered saline (PBS) for 30 min, rinsed with PBS, attached to poly-*L*-lysine-coated slides, permeabilized with 1% Triton X-100, and blocked with 1% Bovine Serum Albumin (Sigma-Aldrich). For the visualization of axonemes, primary mouse monoclonal anti-α-tubulin and secondary anti-mouse IgG-TRITC goat antibodies (1:500 and 1:100, respectively; both from Sigma-Aldrich) were used. After washing with PBS and staining with either DAPI (4′,6′-diamidino-2-phenylindole; Sigma-Aldrich) or SYTO24 Green Fluorescent Nucleic Acid stain (Thermo Fisher Scientific), slides were observed under Leica DM2500 fluorescent microscope. The samples from cultures and midguts of infected flies were prepared and processed for scanning and transmission electron microscopy as described previously [8, 68, 69].

### DNA isolation, amplification, cloning, and sequencing

Total genomic DNA from cultures and one field sample was isolated with the DNeasy Blood & Tissue Kit (Qiagen, Hilden, Germany). 18S rRNA and gGAPDH genes were amplified using the primer pairs S762/S763 [70] and M200/M201 [71], respectively, and sequenced.

### Phylogenetic analyses

The phylogenetic position of the studied trypanosomatids was inferred based on the analyses of 18S rRNA and gGAPDH genes commonly used for this groups as well as more powerful phylogenomic analysis.

Seventy-four 18S rRNA gene sequences of various trypanosomatids including the two species under study (Additional file 11: Table S4) were aligned in MAFFT v. 7.450 using the E-INS-i method [72]. Poorly aligned positions were removed with Gblocks v. 0.91b [73], as described previously [74]. Maximum likelihood (ML) analysis was made in IQTREE v. 1.6.12 [75] under the model SYM + I + G4 rated as the best one by the built-in ModelFinder [76]. Edge support was assessed using 1000 “standard” bootstrap replicates. Bayesian inference was performed in MrBayes v. 3.2.7 [77] under the GTR + I + G model (4 gamma categories) with 2 million generations, sampling frequency set to 200 and other parameters left in default states.

Fifty amino acid sequences of gGAPDH (Additional file 12: Table S5) were aligned in MAFFT using the L-INS-i method and the resulting alignment was trimmed with Gblocks as above. The phylogenetic inference was performed essentially as above, with a few differences specified below. The best amino acid substitution model selected by ModelFinder was LG + I + G4. In MrBayes, the amino acid model prior was set to “mixed” resulting in 1.0 posterior probability of WAG substitution matrix, and it took 3 million generations for the runs to achieve convergence.

The genomic sequences of 26 trypanosomatid species representing all lineages, for which such data are available, were retrieved from the GenBank and TriTryp databases (Additional file 13: Table S6). Inference if orthologous gene groups using OrthoFinder v. 2.2.7 with default settings [78] revealed 624 proteins encoded by single-copy genes. Sequence alignment and trimming were performed using MUSCLE v. 3.8.31 [79] and TrimAl v. 1.2rev59 with the “-strict” option, respectively [80]. The final 45,802 aa-long dataset of 160 protein-coding genes was obtained after applying the following thresholds: minimum 60% average sequence identity as well as maximum 40% and 10% of gaps before and after the trimming, respectively. ML analysis was performed in IQTREE using the LG + C60 + F + G4 with PMSF approximation [81] and edge support estimated by 1000 standard bootstrap replicates. Bayesian inference was carried out in PhyloBayes-MPI v. 1.8 with the LG + CAT + F + G4 model with two parallel chains run for 17,000 iterations [82]. The maximum clade credibility tree was obtained by summarizing all sampled trees but the first 200 removed as burn-in.

### Analysis of cell motility

Cells of the 3–4-day-old S13 culture were transferred to non-coated microscopic slides, covered with 24 × 24 mm coverslips, and examined with 40× objective. This magnification ensured observation of moving parasites for a reasonable time and an opportunity to discriminate the following two categories of cells: (i) with two separate flagella and (ii) with a single or two joint flagella. Videos were recorded at a 720 × 480 pixels resolution with frame rate of 30 fps and then cut into 100 (50 per cell category) 3-s clips for each cell to be analyzed. The cells were selected according to the following criteria: (i) moving actively; (ii) showing no signs of cytotomy, abnormal size, or cell body deformation; and (iii) appearing in the examination area for at least 3 s. The frame-wise coordinates of each cell central point were obtained with the Manual Tracking plugin in Fiji software and used to calculate the following parameters: (i) average speed; (ii) maximum displacement, i.e., maximum distance between any two points on the trajectory; (iii) speed standard deviation (SD); and (iv) relative speed SD, i.e., SD of speed changes expressed as a percentage of an average value. Since all obtained per-category distributions significantly differed from normal as judged by *χ*^2^ test (Additional file 6: Table S2), Mann-Whitney *U* test and Wald-Wolfowitz runs test were used for their comparisons.

## Supplementary information

**Additional file 1: Figure S1.** Cells on a Giemsa-stained smear from the xenic culture F72. **a** Cell appearing as monoflagellate. **b** Unambiguously biflagellate cell.

**Additional file 2: Table S1.** Measurements (in μm) of trypanosomatid cells in three cultures (*N* = 50). A-N and A-K are the distances between the anterior end of the cell and nucleus or kinetoplast, respectively.

**Additional file 3: Figure S2.** Growth dynamics of the cultures CP021 and S13 with values averaged from six independent biological replicates. Confidence bars indicate standard deviations.

**Additional file 4: Figure S3.** Maximum likelihood phylogenetic tree based on gGAPDH gene sequences. Numbers at nodes indicate posterior probability and bootstrap percentage, respectively. Values below 0.5 or 50% are replaced with dashes or omitted. The tree is rooted with the sequence of *Paratrypanosoma confusum*. All well-supported clades of described subfamilies or genera are collapsed. The scale bar denotes number of substitutions per site. The two species of biflagellate trypanosomatids studied here are highlighted.

**Additional file 5: Figure S4.** Flagellates of the strain S13 in the gut (SEM). Abbreviations: tr, trypanosomatids; pm, peritrophic membrane. Scale bar: 20 μm.

**Additional file 6: Table S2.** Summary statistics for motility parameters in two cell categories and results of statistical tests.

**Additional file 7: Table S3.** Correlation of motility parameters in two cell categories.

**Additional file 8: Figure S5:** Three-second trajectories of the cells of the category 1.

**Additional file 9: Figure S6:** Three-second trajectories of the cells of the category 2.

**Additional file 10: Figure S7.** Individually isolated flies in infection longevity experiments.

**Additional file 11: Table S4.** Sequences of 18S rRNA gene used in this work.

**Additional file 12: Table S5.** Sequences of gGAPDH gene used in this work.

**Additional file 13: Table S6.** Genomic sequences used in this work.

## Data Availability

The sequences obtained in this study were submitted to GenBank and are available under accession numbers MT241902 – MT241904 (18S rRNA gene) and MT248930 – MT248932 (gGAPDH gene). The accession numbers of other sequences used in phylogenetic analyses are listed in the supplementary tables. Two datasets were placed to the Figshare depository: (i) for the analysis of the cell cycle [83] doi: 10.6084/m9.figshare.13154483 and (ii) for the motility analysis [84] doi: 10.6084/m9.figshare.13154378.

## Abbreviations

DAPI: 4′,6′-Diamidino-2-phenylindole
FBS: Fetal bovine serum
gGAPDH: Glycosomal glyceraldehyde-3-phosphate dehydrogenase
kDNA: Kinetoplast DNA
ML: Maximum likelihood
PBS: Phosphate-buffered saline
SD: Standard deviation
