# Supplementary figures and images for: Vickermania gen. nov., trypanosomatids that use two joined flagella to resist midgut peristaltic flow within the fly host
Source: BMC Biol. 2020 Dec 2;18:187. doi: 10.1186/s12915-020-00916-y (PMC7712620; doi:10.1186/s12915-020-00916-y)

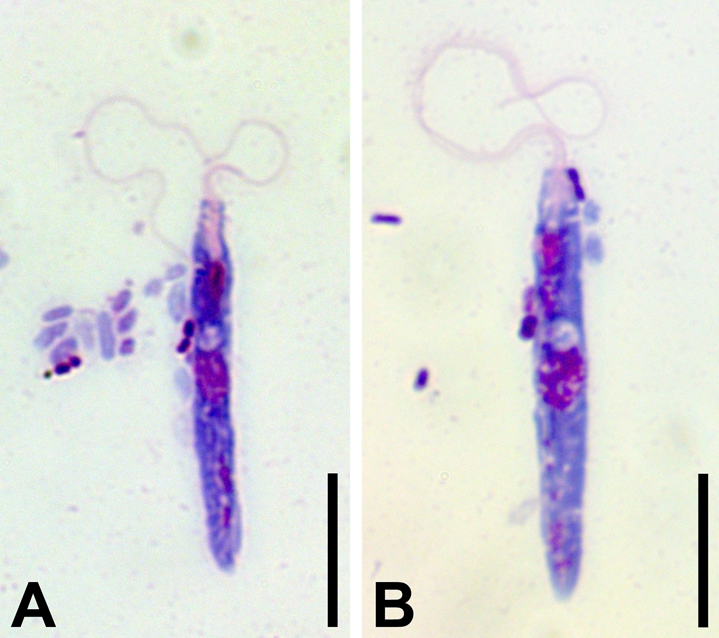

Supplement: Supplementary file 1 — Additional file 1: Figure S1. Cells on a Giemsa-stained smear from the xenic culture F72. a Cell appearing as monoflagellate. b Unambiguously biflagellate cell. [file 12915_2020_916_MOESM1_ESM.tif]

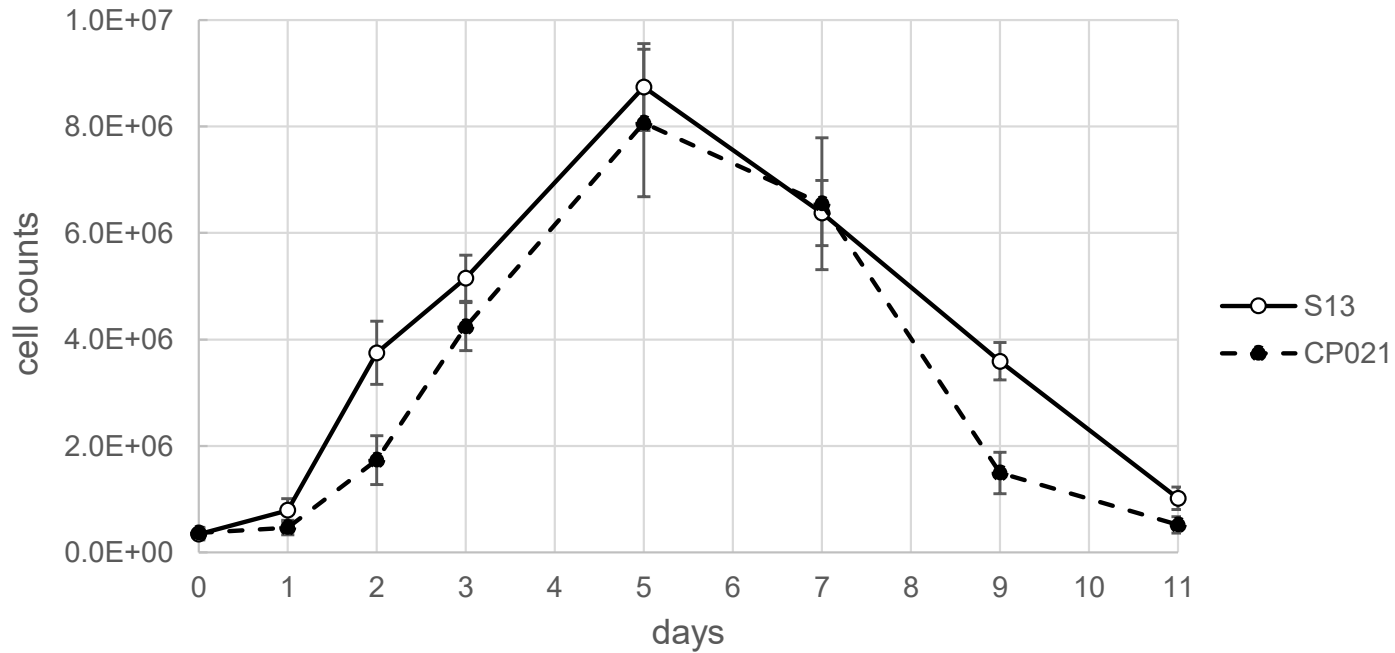

Supplement: Supplementary file 3 — Additional file 3: Figure S2. Growth dynamics of the cultures CP021 and S13 with values averaged from six independent biological replicates. Confidence bars indicate standard deviations. [file 12915_2020_916_MOESM3_ESM.pdf]

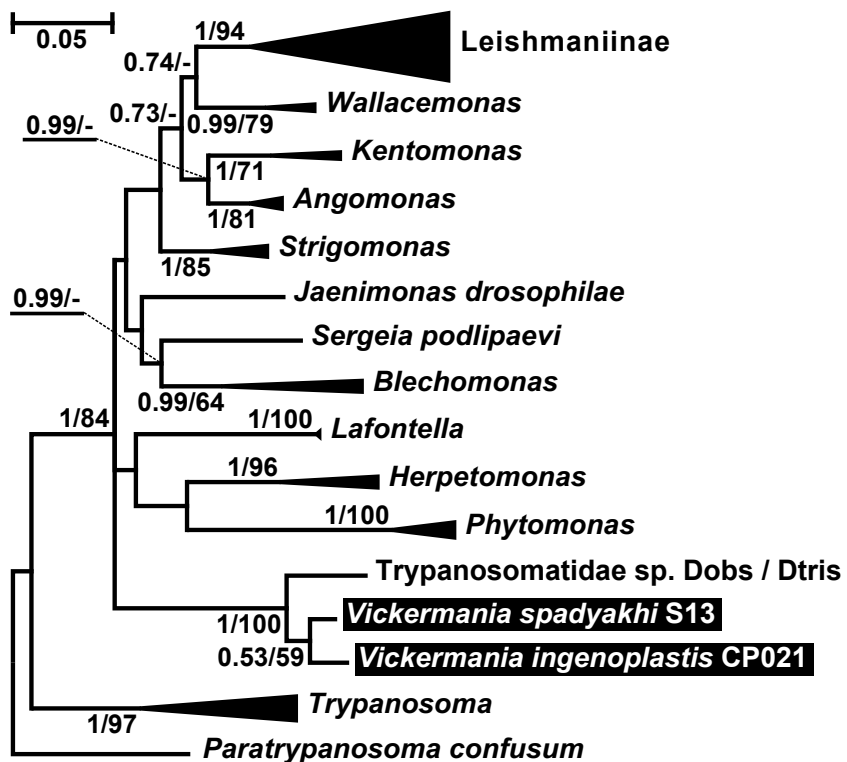

Supplement: Supplementary file 4 — Additional file 4: Figure S3. Maximum likelihood phylogenetic tree based on gGAPDH gene sequences. Numbers at nodes indicate posterior probability and bootstrap percentage, respectively. Values below 0.5 or 50% are replaced with dashes or omitted. The tree is rooted with the sequence of Paratrypanosoma confusum. All well-supported clades of described subfamilies or genera are collapsed. The scale bar denotes number of substitutions per site. The two species of biflagellate trypanosomatids studied here are highlighted. [file 12915_2020_916_MOESM4_ESM.pdf]

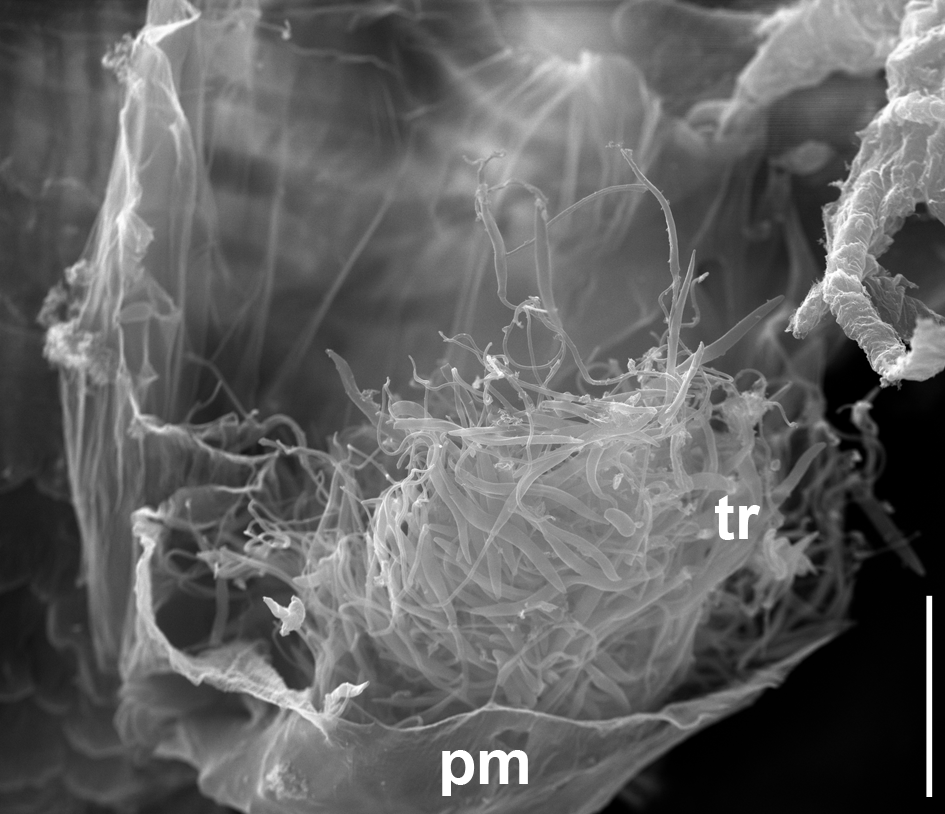

Supplement: Supplementary file 5 — Additional file 5: Figure S4. Flagellates of the strain S13 in the gut (SEM). Abbreviations: tr, trypanosomatids; pm, peritrophic membrane. Scale bar: 20 μm. [file 12915_2020_916_MOESM5_ESM.tif]

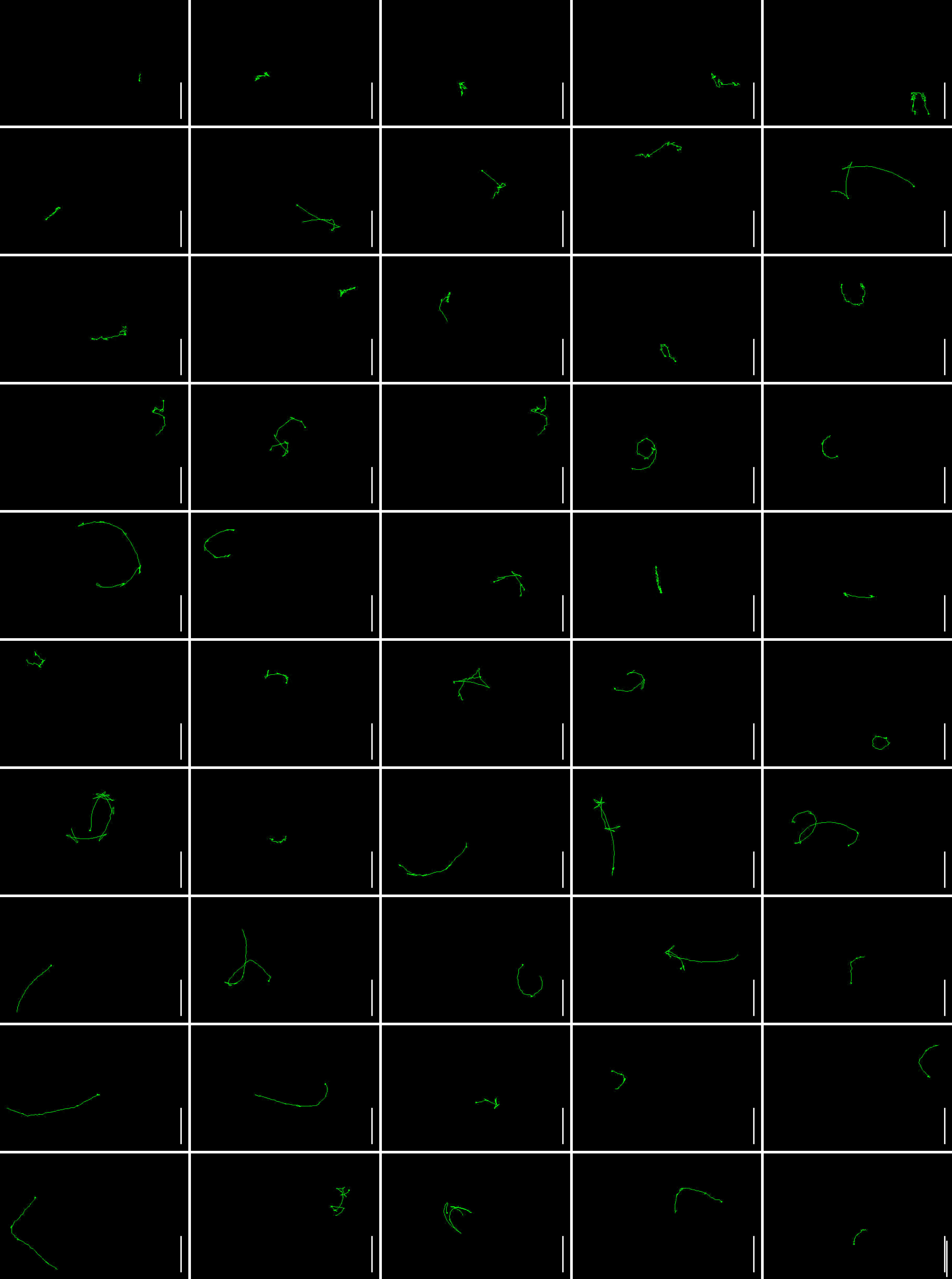

Supplement: Supplementary file 8 — Additional file 8: Figure S5: Three-second trajectories of the cells of the category 1. [file 12915_2020_916_MOESM8_ESM.png]

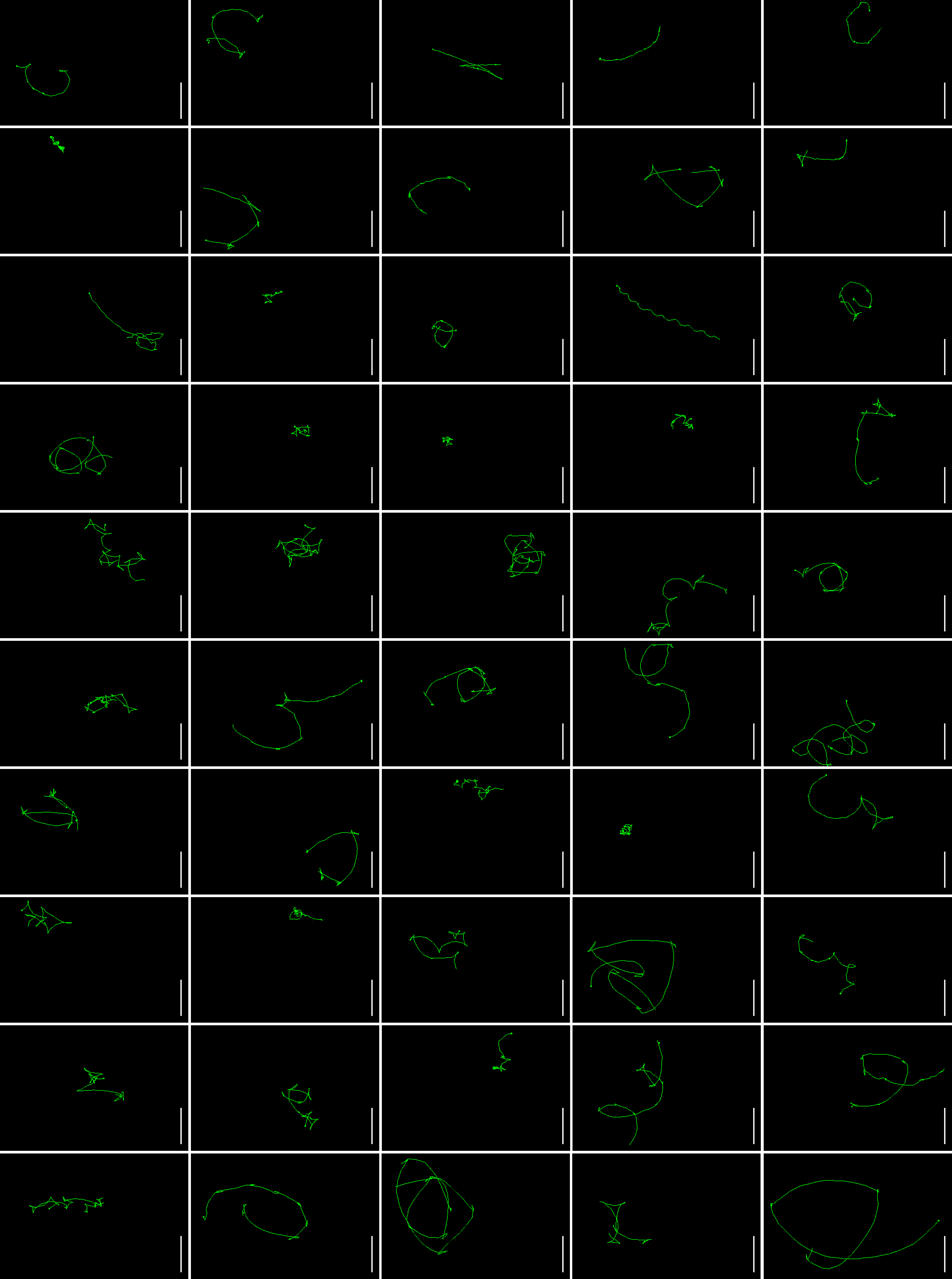

Supplement: Supplementary file 9 — Additional file 9: Figure S6: Three-second trajectories of the cells of the category 2. [file 12915_2020_916_MOESM9_ESM.png]

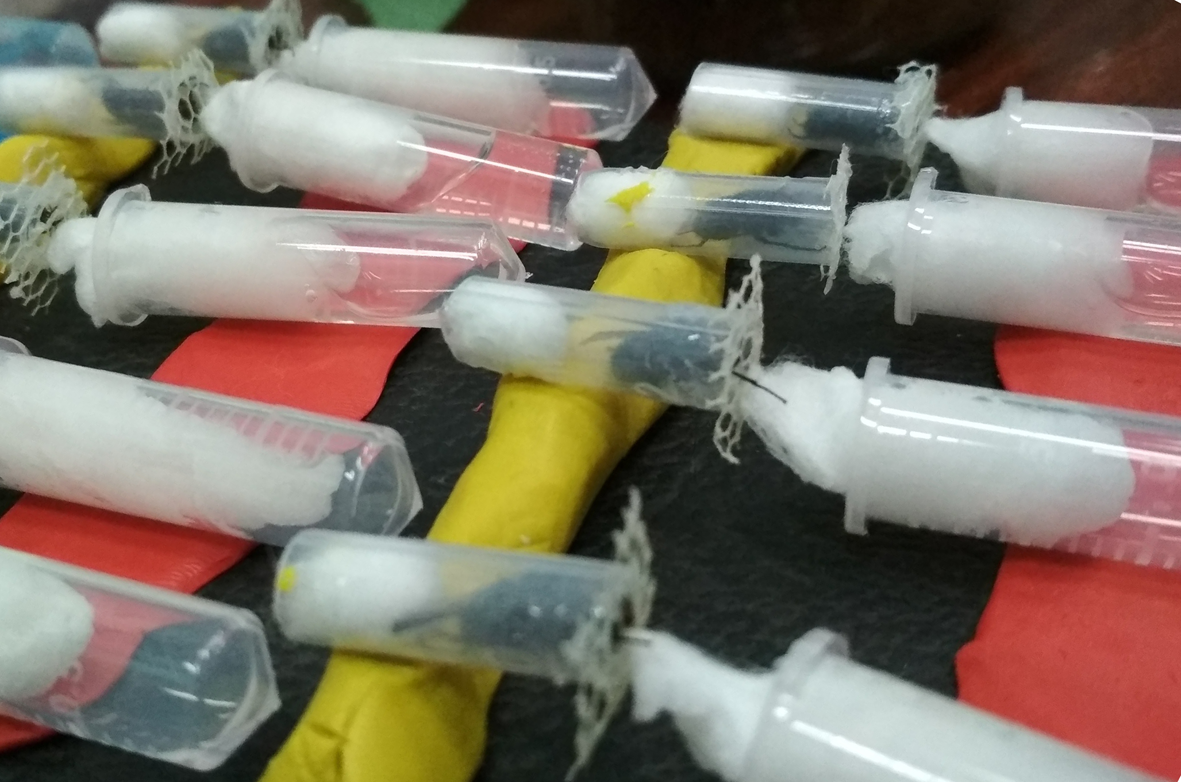

Supplement: Supplementary file 10 — Additional file 10: Figure S7. Individually isolated flies in infection longevity experiments. [file 12915_2020_916_MOESM10_ESM.tif]
