# Supplementary material for: Vickermania gen. nov., trypanosomatids that use two joined flagella to resist midgut peristaltic flow within the fly host
Source: BMC Biol. 2020 Dec 2;18:187. doi: 10.1186/s12915-020-00916-y (PMC7712620; doi:10.1186/s12915-020-00916-y)
Supplement: Supplementary file 2 — Additional file 2: Table S1. Measurements (in μm) of trypanosomatid cells in three cultures (N = 50). A-N and A-K are the distances between the anterior end of the cell and nucleus or kinetoplast, respectively. [file 12915_2020_916_MOESM2_ESM.docx]

| **strain** | **Cell length** | **Cell width** | **Nucleus** | **A-K** | **A-N** | **Flagellum** |
| --- | --- | --- | --- | --- | --- | --- |
| S13 | 34.9 ± 10.4  (22.5 – 75.9) | 2.7 ± 0.6 (1.8 – 4.7) | 4.1 ± 1.0 (2.4 – 7.0) | 3.7 ± 0.9 (2.4 – 7.9) | 12.5 ± 3.4 (8.1 – 22.7) | 38.9 ± 7.5 (25.6 – 64.0) |
| CP021 | 33.3 ± 4.0 (27.3 – 40.7) | 2.8 ± 0.3 (2.3 – 3.5) | 4.1 ± 0.8 (2.6 – 6.0) | 5.5 ± 1.0 (1.4 – 7.4) | 14.4 ± 2.3 (8.1 – 18.6) | 36.8 ± 3.9 (28.8 – 44.5) |
| F72 | 28.2 ± 5.5 (18.9 – 39.3) | 2.4 ± 0.6 (1.7 – 3.7) | 3.9 ± 1.7 (1.0 – 7.8) | 3.9 ± 0.9 (1.8 – 5.5) | 13.2 ± 3.7 (7.9 – 19.9) | 31.3 ± 7.4 (19.9 – 44.9) |
