# Supplementary material for: Vickermania gen. nov., trypanosomatids that use two joined flagella to resist midgut peristaltic flow within the fly host
Source: BMC Biol. 2020 Dec 2;18:187. doi: 10.1186/s12915-020-00916-y (PMC7712620; doi:10.1186/s12915-020-00916-y)
Supplement: Supplementary file 6 — Additional file 6: Table S2. Summary statistics for motility parameters in two cell categories and results of statistical tests. [file 12915_2020_916_MOESM6_ESM.docx]

| Category | Mean | Median | Min | Max | SD | χ2 test for normality *p*-value | Mann-Whithey U test *p*-value | Wald-Wolfowitz runs test *p*-value |
| --- | --- | --- | --- | --- | --- | --- | --- | --- |
| Average speed | | | | | | | | |
| 1 | 52.4 | 47.9 | 8.1 | 131.8 | 24.1 | **<1.0E-05** | **1.4E-11** | **5.7E-08** |
| 2 | 116.6 | 110.4 | 42.3 | 289.1 | 52.8 | **4.6E-04** |  |  |
| Maximum displacement | | | | | | | | |
| 1 | 55.2 | 45.8 | 10.4 | 132.4 | 30.2 | **<1.0E-05** | **7.3E-06** | **6.3E-04** |
| 2 | 93.1 | 87.3 | 16.6 | 239.1 | 45.0 | **1.6E-02** |  |  |
| speed SD | | | | | | | | |
| 1 | 35.2 | 31.3 | 8.4 | 108.4 | 20.8 | **<1.0E-05** | **6.8E-11** | **3.8E-06** |
| 2 | 79.8 | 84.2 | 27.4 | 166.5 | 33.9 | **1.0E-05** |  |  |
| relative speed SD | | | | | | | | |
| 1 | 63.4 | 70.0 | 7.4 | 117.5 | 34.5 | **7.2E-03** | 0.93 | **1.3E-03** |
| 2 | 70.9 | 67.8 | 40.9 | 102.0 | 14.1 | **9.0E-05** |  |  |

Significant *p*-values are shown in bold.
