# Supplementary material for: Vickermania gen. nov., trypanosomatids that use two joined flagella to resist midgut peristaltic flow within the fly host
Source: BMC Biol. 2020 Dec 2;18:187. doi: 10.1186/s12915-020-00916-y (PMC7712620; doi:10.1186/s12915-020-00916-y)
Supplement: Supplementary file 7 — Additional file 7: Table S3. Correlation of motility parameters in two cell categories. [file 12915_2020_916_MOESM7_ESM.docx]

|  | average speed | maximum displacement | speed SD | relative  speed SD |
| --- | --- | --- | --- | --- |
| Category 1 | | | | |
| average speed |  | 0.569 | 0.017 | 0.053 |
| maximum displacement | **<0.001** |  | 0.195 | 0.046 |
| speed SD | 0.907 | 0.175 |  | 0.440 |
| relative speed SD | 0.712 | 0.749 | **0.001** |  |
| Category 2 | | | | |
| average speed |  | 0.608 | 0.584 | -0.248 |
| maximum displacement | **<0.001** |  | 0.213 | -0.320 |
| speed SD | **<0.001** | 0.138 |  | 0.067 |
| relative speed SD | 0.082 | **0.024** | 0.644 |  |

Pearson correlation coefficients and their *p*-values are shown above and below the diagonal, respectively. Significant *p*-values are shown in bold.
