# Supplementary material for: Vickermania gen. nov., trypanosomatids that use two joined flagella to resist midgut peristaltic flow within the fly host
Source: BMC Biol. 2020 Dec 2;18:187. doi: 10.1186/s12915-020-00916-y (PMC7712620; doi:10.1186/s12915-020-00916-y)
Supplement: Supplementary file 11 — Additional file 11: Table S4. Sequences of 18S rRNA gene used in this work. [file 12915_2020_916_MOESM11_ESM.docx]

| **Species** | **Accession number** | **in a collapsed clade** |
| --- | --- | --- |
| *"Leptomonas" jaculum* | EF184218 | - |
| *Angomonas ambiguus* | HM593015 | Strigomonadinae |
| *Angomonas deanei* | HM593011 | Strigomonadinae |
| *Angomonas desouzai* | HM593016 | Strigomonadinae |
| *Blastocrithidia cyrtomeni* | FJ916992 | *Blastocrithidia* |
| *Blastocrithidia largi* | FJ968531 | *Blastocrithidia* |
| *Blastocrithidia miridarum* | KX138600 | *Blastocrithidia* |
| *Blastocrithidia papi* | FJ968531 | *Blastocrithidia* |
| *Blastocrithidia triatomae* | KX138599 | *Blastocrithidia* |
| *Blechomonas campbelli* | KF054133 | *Blechomonas* |
| *Blechomonas keelingi* | KF054130 | *Blechomonas* |
| *Blechomonas luni* | KF054115 | *Blechomonas* |
| *Blechomonas maslovi* | KF054122 | *Blechomonas* |
| *Blechomonas pulexsimulantis* | KF054128 | *Blechomonas* |
| *Borovskyia barvae* | FJ968532 | Leishmaniinae |
| *Crithidia abscondita* | EU079126 | Leishmaniinae |
| *Crithidia brachyflagelli* | JF717840 | Leishmaniinae |
| *Crithidia brevicula* | KJ443344 | Leishmaniinae |
| *Crithidia dedva* | JN624299 | Leishmaniinae |
| *Crithidia fasciculata* | Y00055 | Leishmaniinae |
| *Crithidia insperata* | EU079125 | Leishmaniinae |
| *Crithidia otongatchiensis* | KC205989 | Leishmaniinae |
| *Crithidia permixta* | EU079127 | Leishmaniinae |
| *Crithidia pragensis* | KC205988 | Leishmaniinae |
| *Crithidia* sp. ZM | EU079128 | Leishmaniinae |
| *Crithidia thermophila* | JF717837 | Leishmaniinae |
| *Herpetomonas costoris* | JQ359728 | Phytomonadinae |
| *Herpetomonas muscarum* | JQ359731 | Phytomonadinae |
| *Herpetomonas nabiculae* | JN624300 | Phytomonadinae |
| *Herpetomonas samuelpessoai* | JQ359718 | Phytomonadinae |
| *Jaenimonas drosophilae* | KP260534 | - |
| *Lafontella mariadeanei* | JQ359714 | Phytomonadinae |
| *Leishmania braziliensis* | JX030135 | Leishmaniinae |
| *Leishmania donovani* | CP029526 | Leishmaniinae |
| *Leishmania tarentolae* | M84225 | Leishmaniinae |
| *Leptomonas acus* | DQ910923 | Leishmaniinae |
| *Leptomonas bifurcata* | DQ910925 | Leishmaniinae |
| *Leptomonas jaderae* | EU079123 | Leishmaniinae |
| *Leptomonas moramango* | KC205990 | Leishmaniinae |
| *Leptomonas neopamerae* | DQ910924 | Leishmaniinae |
| *Leptomonas podlipaevi* | DQ383649 | Leishmaniinae |
| *Leptomonas pyrrhocoris* | JN036653 | Leishmaniinae |
| *Leptomonas scantii* | JN036654 | Leishmaniinae |
| *Leptomonas seymouri* | KP717894 | Leishmaniinae |
| *Leptomonas spiculata* | JF717838 | Leishmaniinae |
| *Leptomonas tarcoles* | EF546786 | Leishmaniinae |
| *Leptomonas tenua* | JF717839 | Leishmaniinae |
| *Paratrypanosoma confusum* | KF963538 | - |
| *Phytomonas nordicus* | KT223609 | Phytomonadinae |
| *Phytomonas serpens* | AF016320 | Phytomonadinae |
| *Sergeia podlipaevi* | DQ394362 | - |
| *Strigomonas culicis* | HM593009 | Strigomonadinae |
| *Strigomonas galati* | HM593010 | Strigomonadinae |
| *Strigomonas oncopelti* | AF038025 | Strigomonadinae |
| *Trypanosoma boissoni* | U39580 | *Trypanosoma* |
| *Trypanosoma brucei* | M12676 | *Trypanosoma* |
| *Trypanosoma cruzi* | FJ900239 | *Trypanosoma* |
| *Trypanosoma granulosum* | AJ620551 | *Trypanosoma* |
| *Trypanosoma mega* | AJ223567 | *Trypanosoma* |
| *Trypanosoma pestanai* | AJ009159 | *Trypanosoma* |
| Trypanosomatidae sp. 6.2 | KC183712 | - |
| Trypanosomatidae sp. D44-1 | MG845924 | - |
| Trypanosomatidae sp. G42 | JQ658831 | - |
| Trypanosomatidae sp. GMO-05 | KC205998 | - |
| Trypanosomatidae sp. MCC-01 | MK056196 | - |
| Trypanosomatidae sp. MCC-02 | MK056195 | - |
| Trypanosomatidae sp. MCC-03 | MK056197 | - |
| Trypanosomatidae sp. S13e | MT241902 | - |
| *Vickermania ingenoplastis* | MT241904 | - |
| *Vickermania spadyakhi* | MT241903 | - |
| *Wallacemonas collosoma* | AF153038 | *Wallacemonas* |
| *Wallacemonas raviniae* | KC205996 | *Wallacemonas* |
| *Wallacemonas rigidus* | JN582047 | *Wallacemonas* |
| *Zelonia costaricensis* | KX790782 | Leishmaniinae |
