# Supplementary material for: Vickermania gen. nov., trypanosomatids that use two joined flagella to resist midgut peristaltic flow within the fly host
Source: BMC Biol. 2020 Dec 2;18:187. doi: 10.1186/s12915-020-00916-y (PMC7712620; doi:10.1186/s12915-020-00916-y)
Supplement: Supplementary file 12 — Additional file 12: Table S5. Sequences of gGAPDH gene used in this work. [file 12915_2020_916_MOESM12_ESM.docx]

**Table S3** Sequences of gGAPDH gene used in this work.

| **Species** | **Accession number** | **in a collapsed clade** |
| --- | --- | --- |
| *Angomonas ambiguus* | AVK77716 | *Angomonas* |
| *Angomonas deanei* | EPY36499 | *Angomonas* |
| *Angomonas desouzai* | AVK77713 | *Angomonas* |
| *Blechomonas campbelli* | AGO36718 | *Blechomonas* |
| *Blechomonas luni* | AGO36715 | *Blechomonas* |
| *Blechomonas pulexsimulantis* | AGO36711 | *Blechomonas* |
| *Borovskyia barvae* | ACR54265 | Leishmaniinae |
| *Crithidia brevicula* | AIK25532 | Leishmaniinae |
| *Crithidia fasciculata* | G3PG_CRIFA | Leishmaniinae |
| *Crithidia mellificae* | AIF30811 | Leishmaniinae |
| *Crithidia thermophila* | ARH02611 | Leishmaniinae |
| *Endotrypanum monterogeii* | APQ47645 | Leishmaniinae |
| *Herpetomonas muscarum* | AAZ72729 | *Herpetomonas* |
| *Herpetomonas samuelpessoai* | AAD02466 | *Herpetomonas* |
| *Herpetomonas tarakana* | ALJ56052 | *Herpetomonas* |
| *Jaenimonas drosophilae* | ALE33692 | - |
| *Kentomonas sorsogonicus* | AJE63447 | *Kentomonas* |
| *Kentomonas* sp. ECU-06 | AJE63449 | *Kentomonas* |
| *Lafontella mariadeanei* | AFT63959 | *Lafontella* |
| *Lafontella* sp. GMO-01 | QBY26437 | *Lafontella* |
| *Leishmania braziliensis* | XP_1566920 | Leishmaniinae |
| *Leishmania donovani* | TPP43236 | Leishmaniinae |
| *Leishmania major* | XP1684903 | Leishmaniinae |
| *Leishmania tarentolae* | AAZ72730 | Leishmaniinae |
| *Leptomonas podlipaevi* | AAY83836 | Leishmaniinae |
| *Leptomonas pyrrhocoris* | AAK32964 | Leishmaniinae |
| *Leptomonas seymouri* | AAD02467 | Leishmaniinae |
| *Lotmaria passim* | AIF30831 | Leishmaniinae |
| *Novymonas esmeraldas* | AMN08928 | Leishmaniinae |
| *Paratrypanosoma confusum* | AGG11496 | - |
| *Phytomonas francai* | AOZ73587 | *Phytomonas* |
| *Phytomonas lipae* | QBY26609 | *Phytomonas* |
| *Phytomonas oxycareni* | QBY26436 | *Phytomonas* |
| *Phytomonas serpens* | ABW81350 | *Phytomonas* |
| *Sergeia podlipaevi* | ABD85039 | - |
| *Strigomonas culicis* | EPY19235 | *Strigomonas* |
| *Strigomonas galati* | AEA30294 | *Strigomonas* |
| *Strigomonas oncopelti* | ABW74512 | *Strigomonas* |
| *Trypanosoma boissoni* | CAF04209 | *Trypanosoma* |
| *Trypanosoma brucei gambiense* | XP_11774215 | *Trypanosoma* |
| *Trypanosoma cruzi* | RNC47213 | *Trypanosoma* |
| *Trypanosoma pestanai* | CAF04239 | *Trypanosoma* |
| *Trypanosoma rotatorium* | CAF04220 | *Trypanosoma* |
| *Trypanosoma theileri* | XP_28884833 | *Trypanosoma* |
| *Trypanosomatidae* sp. Dobs | ADU34196 | - |
| *Vickermania ingenoplastis* | MT248932 | - |
| *Vickermania spadyakhi* | MT248930 | - |
| *Wallacemonas collosoma* | ABW81356 | *Wallacemonas* |
| *Wallacemonas raviniae* | AHG98277 | *Wallacemonas* |
| *Zelonia costaricensis* | ABD49445 | Leishmaniinae |
