# Supplementary material for: Vickermania gen. nov., trypanosomatids that use two joined flagella to resist midgut peristaltic flow within the fly host
Source: BMC Biol. 2020 Dec 2;18:187. doi: 10.1186/s12915-020-00916-y (PMC7712620; doi:10.1186/s12915-020-00916-y)
Supplement: Supplementary file 13 — Additional file 13: Table S6. Genomic sequences used in this work. [file 12915_2020_916_MOESM13_ESM.docx]

**Table S4.** Genomic sequences used in this work.

| **Species** | **Strain** | **Data source** | **Accession/weblink** |
| --- | --- | --- | --- |
| *Paratrypanosoma confusum* | CUL13-MS | TriTrypDB-37 | https://tritrypdb.org/tritrypdb/app/downloads/release-37/PconfusumCUL13/ |
| *Trypanosoma rangeli* | SC58 | TriTrypDB-35 | https://tritrypdb.org/tritrypdb/app/downloads/release-35/TrangeliSC58/ |
| *Trypanosoma cruzi* | CL Brener Esmeraldo-like | TriTrypDB-35 | https://tritrypdb.org/tritrypdb/app/downloads/release-35/TcruziCLBrenerEsmeraldo-like/ |
| *Trypanosoma grayi* | ANR4 | TriTrypDB-35 | https://tritrypdb.org/tritrypdb/app/downloads/release-35/TgrayiANR4/ |
| *Trypanosoma theileri* |  | NCBI | GCA_002087225.1 |
| *Trypanosoma congolense* | IL3000 | TriTrypDB-35 | https://tritrypdb.org/tritrypdb/app/downloads/release-35/TcongolenseIL3000/ |
| *Trypanosoma brucei brucei* | TREU927 | TriTrypDB-35 | https://tritrypdb.org/tritrypdb/app/downloads/release-35/TbruceiTREU927/ |
| *Blechomonas ayalai* | B08-376 | TriTrypDB-35 | https://tritrypdb.org/tritrypdb/app/downloads/release-35/BayalaiB08-376/ |
| *Vickermania ingenoplastis* | CP021 | NCBI | GCA_010157825.1 |
| *Phytomonas* sp. | EM1 | NCBI | GCA_000582765.1 |
| *Phytomonas* sp. | HART1 | NCBI | GCA_000982615.1 |
| *Herpetomonas muscarum* | TCC001E | NCBI | GCA_000482205.1 |
| *Strigomonas oncopelti* | TCC290E | NCBI | GCA_000482165.1 |
| *Strigomonas galati* | TCC219 | NCBI | GCA_000482125.1 |
| *Angomonas desouzai* | TCC079E | NCBI | GCA_000482185.1 |
| *Leptomonas seymouri* | ATCC 30220 | TriTrypDB-35 | https://tritrypdb.org/tritrypdb/app/downloads/release-35/LseymouriATCC30220/ |
| *Leptomonas pyrrhocoris* | H10 | TriTrypDB-35 | https://tritrypdb.org/tritrypdb/app/downloads/release-35/LpyrrhocorisH10/ |
| *Crithidia fasciculata* | CfC1 | TriTrypDB-35 | https://tritrypdb.org/tritrypdb/app/downloads/release-35/CfasciculataCfCl/ |
| *Endotrypanum monterogeii* | LV88 | TriTrypDB-35 | https://tritrypdb.org/tritrypdb/app/downloads/release-35/EmonterogeiiLV88/ |
| *Leishmania panamensis* | MHOM/CO/L81L13 | TriTrypDB-35 | https://tritrypdb.org/tritrypdb/app/downloads/release-35/LpanamensisMHOMCOL81L13/ |
| *Leishmania braziliensis* | MHOM/BR/75/M2903 | TriTrypDB-35 | https://tritrypdb.org/tritrypdb/app/downloads/release-35/LbraziliensisMHOMBR75M2903/ |
| *Leishmania tarentolae* | RTAR/DZ/1939/Parrot-TarII | TriTrypDB-35 | https://tritrypdb.org/tritrypdb/app/downloads/release-35/LtarentolaeParrotTarII/ |
| *Leishmania adleri* | MARV/ET/1975/HO174 | NCBI | GCA_902369305.1 |
| *Leishmania mexicana* | MHOM/GT/2001/U1103 | TriTrypDB-35 | https://tritrypdb.org/tritrypdb/app/downloads/release-35/LmexicanaMHOMGT2001U1103/fasta/data/ |
| *Leishmania major* | MHOM/IL/80/Friedlin; clone V1; Friedlin | TriTrypDB-35 | https://tritrypdb.org/tritrypdb/app/downloads/release-35/LmajorFriedlin/ |
| *Leishmania donovani* | MHOM/NP/2003/BPK282/0cl4; BPK282A1 | TriTrypDB-35 | https://tritrypdb.org/tritrypdb/app/downloads/release-35/LdonovaniBPK282A1/ |
